# RE: Field triage in trauma – do the data really justify the conclusions?

**DOI:** 10.1186/1757-7241-17-25

**Published:** 2009-05-29

**Authors:** Marius Rehn, Torsten Eken, Andreas Jorstad Krüger, Petter Andreas Steen, Nils Oddvar Skaga, Hans Morten Lossius

**Affiliations:** 1 Department of Research and Development, Norwegian Air Ambulance Foundation, Drobak, Norway; 2 Faculty of Medicine, Faculty Division Ulleval University Hospital, University of Oslo, Norway; 3 Department of Anaesthesiology, Aker University Hospital, Oslo, Norway; 4 Department of Anaesthesiology and Emergency Medicine, St Olav University Hospital, Trondheim, Norway; 5 Prehospital division, Ulleval University Hospital, Oslo, Norway; 6 Department of Anaesthesiology, Division of Emergency Medicine, Ulleval University Hospital, Oslo, Norway

## Letter

Dear Sir,

Thank you for your interest in our article; "Precision of field triage in patients brought to a trauma centre after introducing trauma team activation guidelines" [1], which gives us the opportunity for expounding some conclusions that could be open for misinterpretation.

We agree with Dr. Sandberg that paramedics and anaesthetists conduct missions with very skewed profiles. We suspect that this mission-selection bias applies to all anaesthetists-manned services, regardless of transport method. The differences in task profile may be beyond the scope of statistical adjustment contributing to a contra-comparison line of argumentation. This is a problem in most epidemiologic studies. What is found is an association between factors; a good starting point for prospective intervention studies. In this case possibly testing changes in one or more of the links in the triage chain. Hopefully, readers agree with us in our statement "skewed mission profiles make comparison of differences in triage precision difficult".

Dr. Sandberg correctly states that the formal decision to activate the trauma team is not made in-field, but in-hospital by the ED nurse. We still used the term field triage, in an attempt to differentiate the study from those that describe traditional ED triage algorithms. Regardless of where the formal decision is made, triage decisions made before the patient arrives in the ED are based upon information gathered in-field and the triage decision have in-field consequences.

We agree with Dr. Sandberg that it is difficult to isolate the aetiology of over- and undertriage. Over- and undertriage rates reflect a chain of events. We did not attempt to identify the link in this chain with most potential for improvement. This is reflected in our recommended improvement initiatives that address every major link in the trauma triage chain: improved on-scene patient evaluation, better routines in communicating patient data from EMS units to the nurse coordinator in the ED, additional training in triage decision-making for nurse coordinators, and development of a two-tiered trauma triage protocol.

We acknowledge the complexity of describing undertriage in multi-centre trauma systems. Our analysis was limited to patients primary admitted to Ulleval University Hospital and did not include those admitted elsewhere in the trauma system. Although not studying the entire trauma system, the article's main findings support the general tendency of imprecise trauma triage in several Scandinavian studies [2-5]. This trend deserves verification through a Norwegian pan-trauma system analysis facilitated by the hopefully soon-to-be national trauma registry.

A degree of both over- and undertriage is unavoidable, and trauma systems should prepare for handling undertriage most effectively. One possible contribution to effectively identify patients subject to undertriage would be to introduce protocol-based ED triage algorithms as a safety net. By introducing a minor trauma team that systematically evaluates patients with uncertain injury panorama, the hospital acknowledges the difficulty of evaluating patients in-field by lowering the threshold for trauma team activation. This two-tiered system may contribute to lowering the undertriage rate while reducing the impact of overtriage. In our opinion a constructive combination.
